# Seaweed Polysaccharide-Based Nanoparticles: Preparation and Applications for Drug Delivery

**DOI:** 10.3390/polym8020030

**Published:** 2016-01-26

**Authors:** Jayachandran Venkatesan, Sukumaran Anil, Se-Kwon Kim, Min Suk Shim

**Affiliations:** 1 Division of Bioengineering, Incheon National University, Incheon 406-772, Korea; jvenkatesan@inu.ac.kr; 2 Department of Preventive Dental Sciences, College of Dentistry, Jazan University, P.O Box 114, Jazan 45142, Saudi Arabia; drsanil@gmail.com; 3 Marine Bioprocess Research Center and Department of Marine-bio Convergence Science, Pukyong National University, Busan 608-737, Korea

**Keywords:** alginate, carrageenan, fucoidan, drug delivery

## Abstract

In recent years, there have been major advances and increasing amounts of research on the utilization of natural polymeric materials as drug delivery vehicles due to their biocompatibility and biodegradability. Seaweed polysaccharides are abundant resources and have been extensively studied for several biological, biomedical, and functional food applications. The exploration of seaweed polysaccharides for drug delivery applications is still in its infancy. Alginate, carrageenan, fucoidan, ulvan, and laminarin are polysaccharides commonly isolated from seaweed. These natural polymers can be converted into nanoparticles (NPs) by different types of methods, such as ionic gelation, emulsion, and polyelectrolyte complexing. Ionic gelation and polyelectrolyte complexing are commonly employed by adding cationic molecules to these anionic polymers to produce NPs of a desired shape, size, and charge. In the present review, we have discussed the preparation of seaweed polysaccharide-based NPs using different types of methods as well as their usage as carriers for the delivery of various therapeutic molecules (e.g., proteins, peptides, anti-cancer drugs, and antibiotics). Seaweed polysaccharide-based NPs exhibit suitable particle size, high drug encapsulation, and sustained drug release with high biocompatibility, thereby demonstrating their high potential for safe and efficient drug delivery.

## 1. Introduction

Seaweed is an important marine resource for human kind, and in particular, for the multi-billion dollar companies that have been operating based on seaweed-derived polysaccharides for approximately the last six decades [1,2,3,4]. The cell walls of seaweed are mainly composed of polysaccharides. These polysaccharides are generally small sugar units linked with glycosidic bonds. In recent years, significant research has been conducted on seaweed for the production of bioenergy and the development of food applications due to the abundance of this resource [5,6,7,8,9,10,11,12,13]. Applications of diverse seaweed polysaccharides (e.g., alginate, carrageenan, ulvan, and laminarin) in drug delivery, tissue engineering, and biosensor areas have been reported [14]. Recently, particular attention has been directed toward developing drug delivery systems using seaweed polysaccharides, which is an important field of biomedical research. Among the various synthetic and natural polymers that have been extensively studied for biomedical applications, particularly for drug delivery [15,16,17,18,19,20], natural seaweed polysaccharides that have been formulated into nanoparticles (NPs) for drug delivery systems (DDS) will be discussed in this review. Natural polysaccharides for DDS have main advantages in their biocompatibility and charge properties [21]. They are also inexpensive materials due to their abundance [22,23,24].

## 2. Polysaccharide-Based Nanoparticles for Drug Delivery

(C_6_H_5_O_10_)*_n_* is the general formula for typical polysaccharides. The number of units (*n*) can vary from 40 to 3000 [25]. Natural polysaccharides are commonly obtained from several resources, including algae, animals, plants, and microbes. Cellulose, chitin, chitosan, alginate, heparin, hyaluronic acid, chondroitin sulfate, pectin, pullulan, amylose, dextran, ulvan, carrageenan, and their derivatives have been widely studied for several biological and biomedical applications, including those in the fields of tissue engineering, wound management, drug delivery, and biosensors [26,27,28]. Furthermore, polysaccharides can be divided into two groups according to their charge. For example, chitosan is a positively charged (cationic) polysaccharide, whereas alginate, carrageenan, and fucoidan are negatively charged (anionic) polysaccharides [21]. Generally, polysaccharides are considered safe, biocompatible, stable, hydrophilic, and biodegradable, and they can be modified into different forms, such as chemically modified polysaccharides, hydrogels, scaffolds, fibers, and NPs. NPs have many advantages for drug delivery purposes compared with larger (micro-sized) particles because they easily penetrate into targeted areas [29,30,31,32,33,34,35,36,37,38,39].

**Figure 1:**
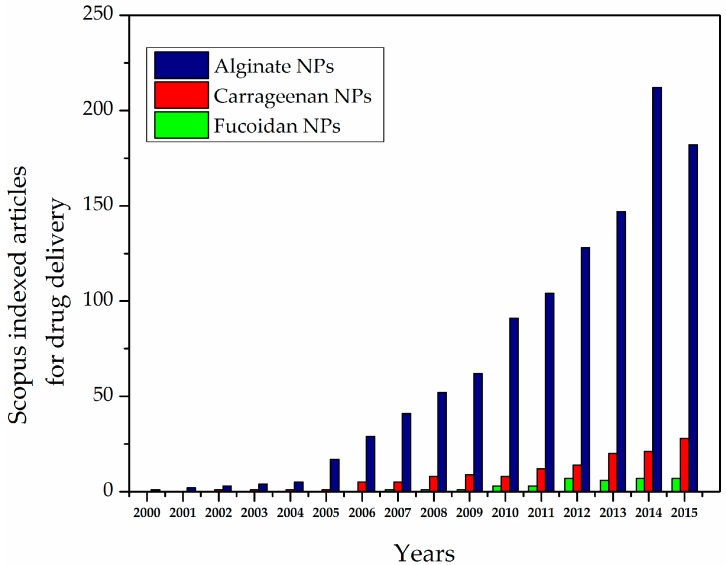
Scopus-indexed articles for alginate-, carrageenan-, and fucoidan-based nanoparticles (NPs) for drug delivery.

Polysaccharide-based NPs can be obtained using different types of methods. In particular, the most widely studied methods are ionic linking, covalent cross-linking, self-assembly, and polyelectrolyte systems. Research on polysaccharide-based NPs (e.g., alginate, carrageenan, and fucoidan) for DDS has been increasing dramatically over the last decade (Figure 1) [21,40]. Polysaccharide-based NPs have advantages due to abundant availability and biocompatible properties, which make them important candidates for drug delivery system [41,42,43,44]. Posocco *et al.* (2015) [45] suggested that polysaccharide-based materials exhibit the following advantages:

- Their sources are abundant and they can be available in a well-characterized state.
- They can be modified to form different materials using chemical and enzymatic methods.
- They are biodegradable and biocompatible and exhibit low immunogenicity.
- They can be useful in stimuli-responsive DDS.
- They can be produced complexed and conjugated with proteins and bioactives.
- They can be modified as gels.
- They can give rise to interpenetrated polymeric networks.
- Ionic polysaccharides are mucoadhesive.

Based on these properties, polysaccharides can be useful as drug delivery carriers.

## 3. Seaweed Polysaccharide-Based Nanoparticles for Drug Delivery

Seaweed can be classified as red, green, or blue. The cell walls of seaweed are often composed of polysaccharides. For approximately four decades, research has been conducted on the structures and applications of seaweed polysaccharides, especially on their functional food applications [46]. Polysaccharides including agar, alginate, fucoidan, carrageenan, and laminarin have been isolated from seaweed [6,25,47].

Seaweed polysaccharides have hydrophilic surface groups, such as hydroxyl, carboxyl, and sulfate groups, which interact with biological tissues easily [48]. Owing to these properties of seaweed polysaccharides, the usage of seaweed polysaccharides in DDS is increasing.

The main difference between the sulfated polysaccharides and other polysaccharides is surface charge. Most of the algae-derived polysaccharides are anionic in nature. Some seaweed-derived polysaccharides have anionic sulfate groups, which are not present in polysaccharides of terrestrial and animal origin [49]. These seaweed polysaccharide-based NPs avoid aggregation during blood circulation by reduced interaction with serum proteins.

## 4. Alginate

**Figure 2:**
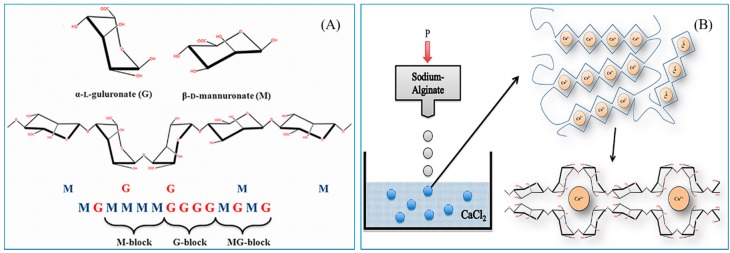
(**A**) The structure of alginate; (**B**) The formulation of egg box-shaped NPs by an ionic gelation method. The figures were adopted with permission from [60].

Alginate is a water soluble, anionic polymer, commonly produced from marine brown algae. It is mainly composed of α-l-guluronic acid (G) and β-d-mannuronic acid (M) residues linked by 1,4-glycosidic linkages (Figure 2A). It is nontoxic, biocompatible, biodegradable, and inexpensive, and thus it is extensively used for several biological, biomedical, and functional food applications [8,50,51]. Alginate NPs can be prepared by different types of methods, including ionic cross-linking, covalent cross-linking, self-assembly, complexation methods, and emulsion methods [39,52,53,54,55,56,57,58,59].

### 4.1. Production of Alginate NPs

Considerable attention has been directed toward preparative methods to produce the desired properties of alginate NPs for effective drug delivery systems [61,62,63]. Different types of methods are explained here.

#### 4.1.1. Ionic Cross-Linked Alginate NPs (Ionotropic Gelation)

The preparation of alginate NPs by ionic gelation is generally simple and mild. They can be produced by cross-linking alginate with various ions, such as Ca^2+^, Ba^2+^, and Al^3+^ [64]. Alginate NPs are commonly formed by the addition of calcium ions at a particular concentration; this is one of the highly explored methods [65]. Ionic cross-linked alginate NPs usually form egg box shapes, as illustrated in Figure 2B. However, sometimes this method tends to produce micro-sized particles rather than NPs. Therefore, process optimization is important to produce alginate NPs of a desired shape. The optimization can be performed by tailoring calcium ion concentration, alginate concentration, addition speed, pH, temperature, and stirring speed.

#### 4.1.2. Preparation of Alginate NPs Using Emulsions

The size of alginate NPs prepared by emulsions is usually below 250 nm. This size is highly desirable for drug delivery applications due to enhanced cellular uptake. Machado *et al.* [66] developed calcium alginate NPs by a water-in-oil (W/O) emulsion. Tetraethylene glycol monododecyl ether, as a nonionic surfactant in decane, was mixed with alginate solution at different concentrations to form emulsions. Then, CaCl_2_ was added into the W/O nanoemulsions to form alginate NPs. Finally, alginate NPs were separated from the aqueous phase. The diameter of the developed NPs was approximately 200 nm [53,56,66,67,68].

#### 4.1.3. Polyelectrolyte Complexation of Alginate NPs

The production of NPs with polyelectrolyte complex (PEC) systems has gained much attention due to its simple procedure for drug delivery applications. Generally, PECs can be formed by mixing oppositely charged polyelectrolytes and allowing them to interact electrostatically [69]. Aqueous polycationic solutions (chitosan or poly-l-lysine) were mixed with polyanionic alginate solutions at room temperature to immediately produce alginate-cationic polymeric NPs [70,71]. pH, temperature, and stirring speed may play major roles in controlling the size of these alginate NPs [72].

### 4.2. Alginate NPs in Drug Delivery Systems

Alginate NPs have been extensively studied for DDS due to their high encapsulation efficiency of highly effective drugs, proteins, and peptides. Alginate NPs usually do not agglomerate in organs while they deliver drugs or proteins [73]. Alginate NPs chemically modified with encapsulation materials may exhibit prolonged periods of material delivery. NP stability is an important parameter in DDS. Azevedo *et al.* [74] developed alginate-chitosan NPs with high stability. They were stored at 4 °C in solution for a period of five months. Their particle size and zeta potential were measured during that period of time. Particle size may change, and they may aggregate over time; this may due to the weak electrostatic interactions between alginate and chitosan. However, the addition of a stabilizer can overcome this type of issue. For example, the addition of vitamin B_2_ maintained the stability of alginate–chitosan NPs over a five-month period of time [74].

#### 4.2.1. Alginate NPs in Protein and Peptide Delivery

Quality of life can be reduced significantly by health problems and common diseases. It was estimated that 9% of adults aged 18+ years and approximately 1.5 million deaths were directly caused by diabetes. The World Health Organization (WHO) predicts that by 2030, diabetes will be the 7th leading cause of death [75,76]. Insulin is one of the main treatments for diabetes, and the bioavailability of oral insulin is limited by the gastrointestinal tract. As a result, the targeted delivery of insulin is a main objective of NP-based insulin delivery. Polymers play an important role in insulin delivery [77]. Table 1 shows the usage of various alginate NPs for protein delivery, such as insulin delivery.

**Table 1:** Alginate NPs for protein drug delivery.

Serial number	Materials	Method	Particle size	Drug	References
1	Alginate–chitosan	Ionotropic and polyelectrolyte complex	800 nm	Insulin	[69]
2	Alginate–chitosan	Ionotropic pre-gelation	100–200 nm	Insulin	[77]
3	Alginate	W/O emulsion	2604 nm	Insulin	[78]
4	Alginate–chitosan	Polyelectrolyte complex	700 nm	Insulin	[79]
5	Alginate–chitosan	Gelification	750 nm	Insulin	[80]
6	Alginate–chitosan–TPP	Ionic gelation	260 to 525 nm	Insulin	[81]
7	Alginate–oligochitosan	W/O in microemulsion	136 nm	BSA	[82]
8	Alginate NPs	Microemulsion	350 nm	BSA	[83]
9	Alginate–chitosan	Gelification	200 nm	BSA	[84]

Reis *et al.* [78] developed alginate NPs using a W/O emulsion method and physical cross-linking with calcium ions; it was demonstrated that calcium ions play an important role in controlling particle size. The mass ratio of calcium ions to alginate was 7% (*w*/*w*). The encapsulation efficiency of insulin in the alginate NPs was more than 71%. The smaller particle size was achieved by adjusting the calcium and alginate solution concentrations; higher encapsulation efficiency and lower insulin release at pH 1.2 were also attained in this way [78]. At higher calcium ion concentrations, there are more calcium ions free to react with the M and G alginate monomers, forming more rigid alginate polymer chains and ultimately allowing sustainable insulin release from the alginate.

Sarmento *et al.* [69] prepared alginate NPs by ionotropic pre-gelation with CaCl_2_ followed by a PEC process with chitosan polysaccharides. The pH and mass ratio of the polymers and calcium ions play crucial roles influencing the NP formation. Approximately 800-nm particle sizes were produced by this method at pH 4.7 with a 6:1 mass ratio of alginate to chitosan. Fourier transform infrared spectroscopy results revealed the efficient encapsulation of insulin in the NPs [69]. In work by the same group, alginate NPs were formed by ionic gelation and used for insulin delivery [79]. *In vivo* results of alginate–chitosan NPs loaded with insulin were obtained from diabetic rats. Orally administered NPs lowered glucose levels by more than 40% at dosages of 50 and 100 IU/kg [80].

**Figure 3:**
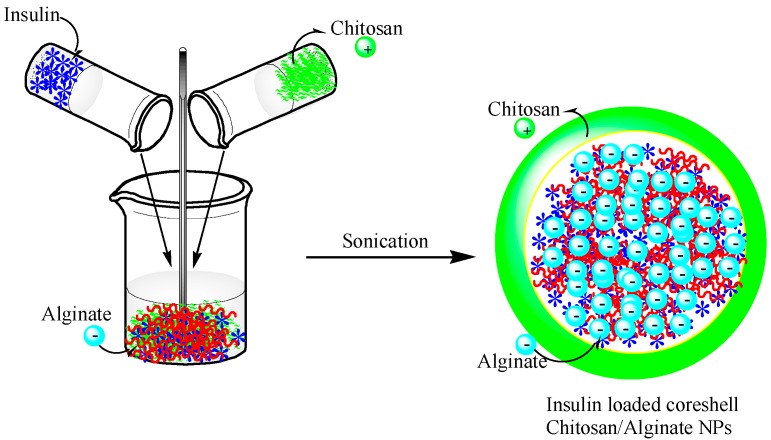
A schematic showing the preparation of chitosan-alginate NPs incorporating insulin. This figure was adopted and redrawn from [77]. Copyright 2015, Elsevier.

The size of the alginate–chitosan NPs was further decreased to less than 250 nm using the same ionotropic pre-gelation method by controlling the polymer mass ratio (Figure 3). The average size of the NPs obtained by this method was approximately 100–200 nm. The encapsulation efficiency of the insulin in the alginate-chitosan NPs was approximately 85%, and sustained release and nontoxicity were observed when the NPs were used as a peroral treatment [77] (Figure 3).

Goycoolea *et al.* [81] developed chitosan–alginate NPs with pentasodium tripolyphosphate (TPP) using ionic gelation and PEC. The particle size was dependent on the molecular weight of alginate. The particle size increased from 260 to 525 nm with increased alginate molecular weight. Insulin was used as a model drug, and the encapsulation efficiency was found range from 41% to 52%. Insulin-loaded chitosan–alginate–TPP NPs showed efficient systemic absorption in rabbits [81].

Alginate-chitosan NPs have been used for the effective delivery of bovine serum albumin (BSA). Wang *et al.* [82] developed NPs based on low molecular weight alginate and chito–oligosaccharides using a microemulsion method. The size of the NPs was approximately 136 nm. The encapsulation efficiency reached approximately 88.4%. The developed NPs were nontoxic, biocompatible, and uniform in size, which suggested that they could be used as vehicles for other drugs [82]. Using the same microemulsion method, alginate NPs were developed using aqueous CaCl_2_, dioctyl sodium sulfosuccinate, and isopropyl myristate. The particle size of the alginate NPs was approximately 350 nm, as measured by DLS. The sustained release of BSA from the alginate NPs was observed. The loading efficiency of BSA was approximately 40% [83]. Li *et al.* [84] developed chitosan–alginate NPs for BSA delivery. The particle size of the NPs was approximately 200 nm. The release of BSA from the NPs was pH dependent [84].

#### 4.2.2. Alginate NPs for Cancer Drug Delivery

Cancer has a major impact on society across the world. The number of new cancer cases will rise to 22 million within the next two decades [85]. Currently, surgery, chemotherapy, and radiation are the main therapies for cancer; however, it has been several years since chemotherapy has been used as the primary treatment for cancer because of the extent to which it can kill normal healthy cells. To overcome this issue, DDS with NPs have become alternative methods of targeting only cancer cell, increasing the availability of drugs to cancer cells and leaving normal cells unaffected [86]. Different types of NPs have been extensively studied for cancer drug delivery. Over the last five decades, liposome-, polymer-, dendrimer-, and protein-based NPs and inorganic NPs have been utilized as drug carriers to treat cancer [87]. NPs based on both synthetic polymers (e.g., poly(lactic-*co*-glycolic acid), polylactic acid, and polycaprolactone) and natural polymers (e.g., alginate, chitosan, carrageenan, and fucoidan) have been used as drug carriers to deliver several cancer drugs, such as doxorubicin and 5-fluorouracil (5-Fu) (Table 2).

**Table 2:** Alginate NPs for cancer drug delivery.

Serial number	Materials	Method	Particle size	Drug	References
1	Alginate	Gelification with CaCl_2_ and poly-l-lysine	250–850 nm	Doxorubicin	[88]
2	Alginate	CaCl_2_ cross-linking	214 ± 11 nm	Doxorubicin	[89]
3	Glycyrrhetinic acid–Alginate NPs	Chemical modification	80 and 100 nm	Doxorubicin	[90]
4	Alginate NPs	Chemical modification	241 nm	Doxorubicin	[91]
5	Aerosol OT-alginate NPs	Emulsification cross-linking method	39 ± 7 nm	Doxorubicin and methylene blue	[92]
6	Alginate–CaCO_3_ NPs	Coprecipitation method	100–400 nm	Doxorubicin and p53	[93,94]
7	Chitosan–alginate NPs	Emulsion method	200 nm	5-Fluorouracil	[95]
8	Alginate–chitosan	Ionic gelation	329–505 nm	5-Fluorouracil	[96]
9	Alginate-chitosan-Pluronic F127	Ionotropic pre gelation	100 ± 20 nm	Curcumin	[97]
10	Alginate NPs	Oligonucleotide/Poly lysine	NA	Antisense oligonucleotide	[98]
11	Alginate–chitosan	Ionotropic gelation method	230 to 627 nm	Gemcitabine	[99]
12	Bovine serum albumin and thiolated alginate	Coacervation	350 to 500 nm	Tamoxifen	[100]

**Figure 4:**
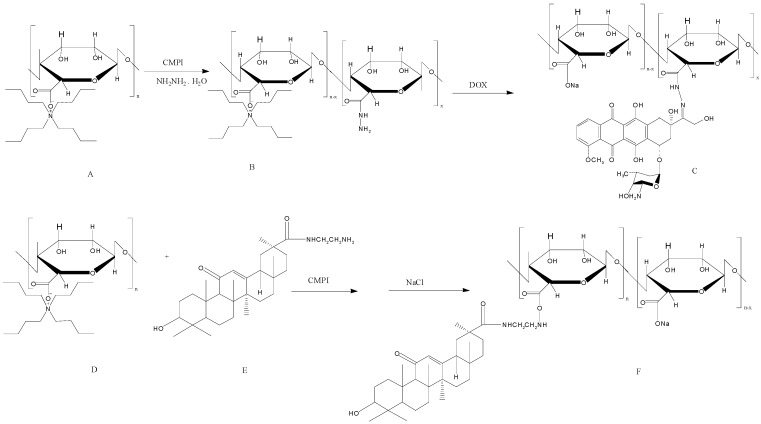
The synthesis route of doxorubicin-modified alginate (DOX–ALG) (**top**) and glycyrrhetinic acid-modified alginate (GA–ALG) (**bottom**). The figures were adopted and redrawn from [91].

**Figure 5:**
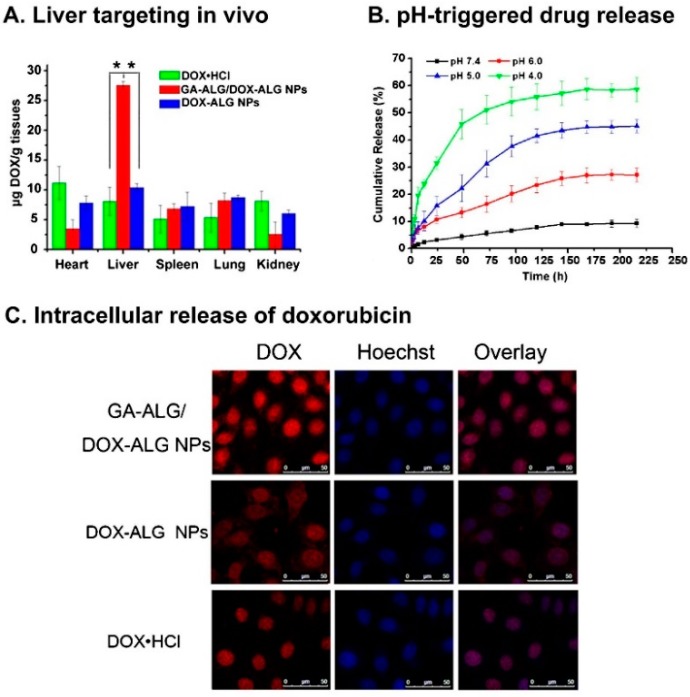

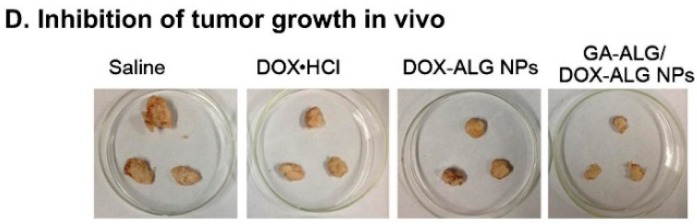
(**A**) The results of an *in vivo* liver targeting study; (**B**) the release results at different pH levels; and (**C**) the cellular uptake of doxorubicin using glycyrrhetinic acid–alginate (GA–ALG)/doxorubicin–alginate (DOX–ALG) NP complexes and doxorubicin–alginate (DOX–ALG) NPs; (**D**) H22 tumor tissue slices from mice treated with saline, doxorubicin, doxorubicin-alginate (DOX–ALG) NPs, and glycyrrhetinic acid-alginate (GA–ALG)/doxorubicin-alginate (DOX–ALG) NP complexes. The figures were adopted and redrawn with permission from [91]. Copyright 2013, Elsevier.

Rajaonarivony *et al.* [88] developed alginate NPs with calcium ions and poly-l-lysine by a gelification method. The particle size of the alginate NPs was approximately 250–850 nm, and they were used for doxorubicin delivery. From this study, significant research has been performed to develop alginate NPs for various drug delivery purposes using a similar type of method [88]. Zhang *et al.* [89] developed alginate NPs with a CaCl_2_ cross-linking method. Alginate was modified with a liver targeting molecule (*i.e.*, glycyrrhetinic acid) and chemically characterized. The doxorubicin-loaded glycyrrhetinic acid-alginate NPs exhibited a size of approximately 214 ± 11 nm. The drug could be released from the NPs for 20 days, and the treatment had the capacity to kill hepatocellular carcinoma cells effectively [89]. The same group examined the *in vivo* therapeutic efficacy of the developed NPs using a mouse liver tumor model. The chemical modification of the alginate NPs with glycyrrhetinic acid increased the biodistribution of doxorubicin. Doxorubicin reached 67.8 ± 4.9 μg/g in the liver after intravenous administration, which was significantly higher compared with the results of both non-glycyrrhetinic acid-modified NPs and the drug only [90]. By the continuous research on complexing NPs, glycyrrhetinic acid-modified alginate (GA–ALG) and doxorubicin-modified alginate (DOX–ALG) were prepared by self-assembly [91] (Figure 4). pH-Sensitive glycyrrhetinic acid–alginate/doxorubicin–alginate NPs (GA-ALG/DOX-ALG NPs) demonstrated efficient treatment of liver cancer. As shown in Figure 5A, DOX concentration in the liver of the GA-ALG/DOX-ALG NPs group reached 27.6 µg/g, which was higher than that of the DOX·HCl (8.1 µg/g). Further, DOX release from GA-ALG/DOX-ALG NPs showed pH-sensitivity; less than 10% of the drugs was released at pH 7.4 within 9 days while 58.7% of drug was released at pH 4.0 (Figure 5B). Confocal laser scanning microscopy images of HepG2 cells incubated with GA-ALG/DOX-ALG NPs and DOX-ALG NPs at the same DOX concentration (10 µg DOX/mL) showed that GA-ALG/DOX-ALG NPs were efficienty taken up by the cells (Figure 5C). H22 tumor tissue treated with GA-ALG/DOX-ALG NPs showed more effective inhibition of tumor growth compared with bare DOX and DOX-ALG NPs (Figure 5D).

Surfactant-polymer hybrid NPs using alginate and an anionic surfactant, aerosol-OT (AOT), were prepared for combined chemotherapy and photodynamic therapy. The NPs were able to deliver both doxorubicin and methylene blue. Increased nuclear and cellular accumulation of doxorubicin and methylene blue enhanced the production of reactive oxygen species that contributed to the superior toxicity [92].

Alginate–calcium carbonate–doxorubicin-p53 NPs were prepared by a co-precipitation technique. p53 is a tumor suppressor gene that plays a pivotal role in DNA repair, apoptosis, and cell cycle regulation. Zhao *et al.* [93,94] stated that, “inhibiting p53 mutations, the reintroduction of wild type (wt) p53 into tumor cells harboring p53 mutations, may also enhance the sensitivity of tumor cells to chemotherapeutic agents through the inhibition of the P-gp expression related to drug resistance. On the other hand, wt p53 protein is positive in response to a variety of stress signals including DNA damage caused by antitumor drugs”. Thus, the combination of p53 and doxorubicin may increase the efficacy of the cancer treatment. The developed particle size, approximately 100 to 400 nm, depended on the polymer content. The NPs showed a high drug encapsulation efficiency and completely inhibited the growth of the HeLa cells. These NPs were used for both gene and drug delivery purposes [93,94]. Xing *et al.* developed chitosan–alginate NPs by an emulsion method to incorporate 5-Fu. 5-Fu is a pyrimidine analog drug that has been used to treat cancer for several decades. The resulting particle size was found to be approximately 200 nm. A drug release of 50% was observed at 12 h *in vitro* [95]. Using the same 5-Fu drug, sodium alginate-chitosan NPs were prepared by an ionic gelation technique. The developed NPs showed a size ranging from approximately 329–505 nm. The encapsulation efficiency of 5-Fu mainly depended on the molar ratios of sodium alginate and chitosan (6%–26%) [96].

Recent studies have reported that curcumin has several biological activities, such as anti-inflammatory, antioxidant, and antimicrobial activity and the inhibition of different types of tumor cells. Das *et al.* [97] developed alginate–chitosan–pluronic F127 NPs for curcumin drug delivery. The encapsulation efficiency of the NPs was improved by the addition of pluronic F127. The size of the NPs was found to be approximately 100 nm [97]. Other studies using alginate NPs for cancer drug delivery have also been reported elsewhere [98,99,100].

#### 4.2.3. Alginate NPs for Antibiotic and Antimicrobial Drug Delivery

Several antimicrobial drugs are available on the market to kill bacteria, viruses, and fungi [101]. Zahoor *et al.* [102] developed alginate NPs as antitubercular drug carriers. Isoniazid, rifampicin, and pyrazinamide were encapsulated by the alginate NPs. The encapsulation efficiency of these drugs was approximately 70%–90%. The size of the alginate NPs was approximately 235.5 nm with a polydispersity index of 0.439 [71,102,103] (Table 3).

Choonara *et al.* (2011) developed alginate NPs with an ionic cross-linking and reverse emulsion method [104]. Ghaffari *et al.* [105] developed alginate–chitosan NPs encapsulating ciprofloxacin with a particle size of approximately 520 ± 16 nm. The loading efficiency of ciprofloxacin was 88%. A sustained release of ciprofloxacin was observed over 45 h [105]. Bi-specific and biodegradable chitosan-alginate polyelectrolyte NPs were developed by Arora *et al.* [72] for amoxicillin delivery. The particle size of the developed NPs was 264 nm. By increasing the chitosan concentration in the polyelectrolyte system, the particle size was increased [72]. Chopra *et al.* [106] developed chitosan–alginate NPs for streptomycin delivery. The size of the developed NPs was 328 nm, and the encapsulation efficiency of the drug was 93.32% [106]. Other alginate-chitosan NPs encapsulating antimicrobial drugs have also been developed [107,108].

**Table 3:** Alginate NPs for antibiotic drug delivery.

Serial number	Materials	Method	Particle Size	Drug	References
1	Alginate NPs	Cation-induced gelification	NA	Rifampicin, isoniazid, pyrazinamide and ethambutol	[71]
2	Alginate–chitosan	Polyelectrolyte complex	264–638 nm	Amoxicillin	[72]
3	Alginate NPs	Cation-induced gelification	235.5 ± 0 nm	Rifampicin	[102]
4	Alginate NPs	Cation-induced gelification	235.5 ± 0 nm	Isoniazid, rifampicin, pyrazinamide, and ethambutol	[103]
5	Alginate	Reverse emulsion	240 ± 8.7 nm	Rifampicin and isoniazid	[104]
6	Calcium alginate	Polyelectrolyte complex	520 nm	Ciprofloxacin	[105]
7	Alginate–chitosan	Ionotropic pre-gelation	328 nm	Streptomycin	[106]
8	Alginate–chitosan–silica	Polyelectrolyte complex	NA	Piperacillin-tazobactam, cefepime, piperacillin, imipenem, gentamicin, ceftazidime	[107]
9	Alginate–chitosan	Gelification	50–250 nm	Nisin	[108]

#### 4.2.4. Alginate NPs for Other Drug Delivery

Alginate NPs are excellent for encapsulating various drugs. Methylene blue, fluorescein sodium salt, nifedipine, gatifloxacin, rhodamine 6G, EGFR phosphorothioated 21-mer antisense 50, turmeric oil, epidermal growth factor, Bupivacaine, vitamin D_3_, 5-aminolevulinic acid, tuftsin, candida rugosa lipase, ibuprofen, ivermectin, enoxaparin, nitric oxide, benzoyl peroxide, and quinapyramine have all been encapsulated in alginate NPs for drug delivery [109,110,111,112,113,114,115,116,117,118,119,120,121,122,123,124,125,126,127,128,129,130,131] (Table 4).

**Table 4:** Alginate NPs for other drug delivery.

Serial number	Materials	Method	Particle size	Drug	References
1	Sodium alginate–chitosan	Ionic gelation, polyelectrolyte	205 to 572 nm	Gatifloxacin	[70]
2	Sodium alginate: CaCl_2_-(poly-l-lysine or chitosan)	Ionic gelation	544 ± 53 nm	Methylene blue	[109]
3	Silica/alginate	NA	50–200 nm		[110]
4	Alginate–chitosan	Ionotropic gelation	600 nm	Fluorescein sodium salt	[111]
5	Alginate–chitosan	Polyelectrolyte	20–50 nm	Nifedipine	[112]
6	OT-alginate hydrogel loaded with Fe_3_O_4_	emulsification-cross-linking process	25 and 50 nm	Rhodamine 6G	[113]
7	Alginate–chitosan	Precipitation method	194 nm	EGFR Phosphorothioated 21-mer antisense 50	[114]
8	Alginate–chitosan	Gelification	522 ± 15 nm	Turmeric oil	[115]
9	Alginate–chitosan	NA	NA	Epidermal growth factor receptor	[116]
10	Alginate–chitosan	Polyelectrolyte	NA	Bupivacaine	[117]
11	Alginate–chitosan	NA	600–650 nm	pAcGFP1-C1 plasmid	[118]
12	Hydrophobic alginate derivative	Chemical modification	200–400 nm,	Vitamin D_3_	[119]
13	Alginate folic acid chitosan	Ionic gelation	115 nm	5-aminolevulinic acid	[120]
14	Alginate NPs	Gelation method	200 nm	Tuftsin	[121]
15	Superparamagnetic sodium alginate NPs	W/O emulsion method	25–30 nm	Candida rugosa lipase	[122]
16	superparamagnetic alginate NPs	Coprecipitation	200 nm	Ibuprofen	[123]
17	Thiolated chitosan alginate	NA	265.7 ± 7.4 to 471.0 ± 6.4 nm	Ocular drug	[124]
18	Chitosan–alginate NPs	Coacervation	155 nm	Ivermectin	[125]
19	Chitosan–alginate NPs	Ionic gelation	213 nm	Enoxaparin	[126]
20	Chitosan–alginate NPs	NA	NA	Nitric oxide	[127]
21	Chitosan–alginate NPs	Polyelectrolyte complex	50 nm	Benzoyl peroxide	[128]
22	Alginate beads	W/O emulsion	200 to 1,000 nm	NA	[129]
23	Alginate	NA	NA	Pesticide	[130]
24	Sodium alginate NPs	Emulsion-cross-linking technology	60 nm	Quinapyramine	[131]

### 4.3. Alginate NP Patents

There are several patents regarding alginate-based NPs with different types of preparative methods. The methods of W/O emulsion and ionic cross-linking with calcium ions are patented [132]. Aerosol alginate NPs with doxorubicin, verapamil, and clonidine are also patented [133].

## 5. Carrageenan NPs

**Figure 6:**
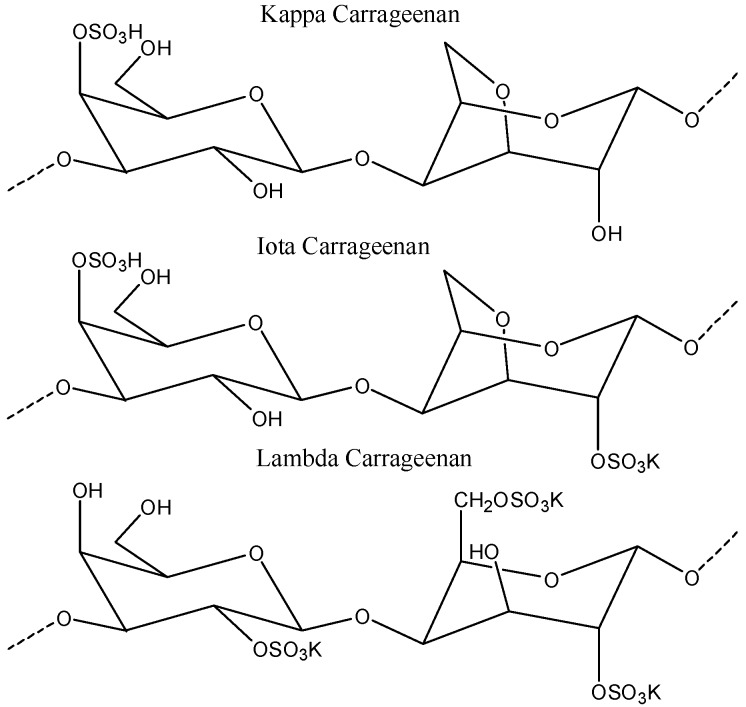
The structure of κ carrageenan, ι carrageenan, and λ carrageenan. The figures were adopted and redrawn from [146].

Carrageenan is an anionic, sulfated polysaccharide and is commonly isolated from red seaweed. It is mainly composed of d-galactose and 3,6-anhydro-d-galactose with glyosidic units. Carrageenan has been widely used for functional food applications and cancer treatments [134,135,136,137,138]. Recently, carrageenan has also been used for several biomedical applications [139,140,141,142,143], which were intensively reviewed by Li *et al.* [144]. The extraction procedure, structure, and subsequent product applications have also been discussed by Prajapati *et al.* (2014) in detail [22,145]. Three different types of carrageenan are available, depending on the extraction procedure: kappa (κ), iota (ι), and lamda (λ) carrageenan [146] (Figure 6).

### 5.1. Production of Carrageenan NPs

**Figure 7:**
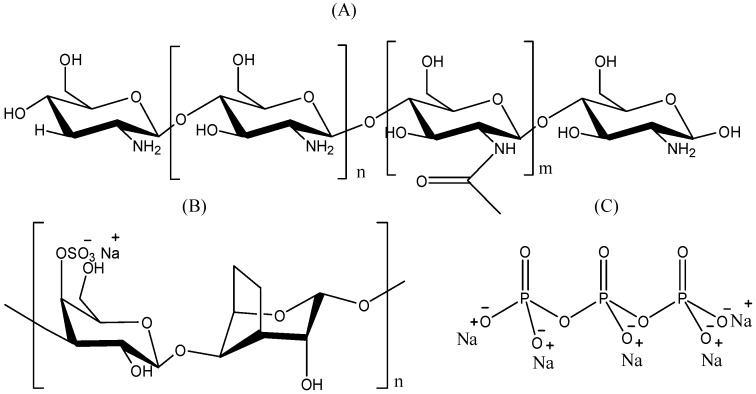
Structure of (**A**) chitosan; (**B**) carrageenan; and (**C**) tripolyphosphate (TPP). The figures were adopted and redrawn from [149].

The negative surface charge of carrageenan can form a PEC with positively charged ion molecules. NPs formed by chitosan-carrageenan complexing have been studied for drug delivery purposes. These NPs can be prepared by the ionic gelation or polyelectrolyte complexing methods by mixing carrageenan with cationic polymers such as chitosan [147] (Figure 7).

Long-term NP stability is a major challenge of polysaccharide-based NPs used for DDS. Rodrigues *et al.* [148] reported chitosan-carrageenan NPs that were developed using a simple polyelectrolyte complexation method. The developed NPs were stored at 4 °C in an aqueous solution, and their size and zeta potential were measured. No statistically significant changes were observed in the size and zeta potential. This indicated that the stability of the NPs was not dependent on the mass ratio of polymers [148]. In work from the same group, the addition of TPP to the chitosan-carrageenan mixture was observed to increase the stability of the NPs for over 250 days [149], suggesting that TPP can act as an effective stabilizer.

### 5.2. Carrageenan NPs as Drug Delivery Vehicles

The most widely used method to prepare carrageenan NPs is the polyelectrolyte method, which is very simple and requires mild conditions. In recent years, particular attention has been directed toward carrageenan-chitosan NPs for the delivery of drug molecules (Table 5). A very mild, feasible, and convenient polyelectrolyte method for the production of carrageenan–chitosan NPs was investigated [150]. Bulger *et al.* [151] developed chitosan-carrageenan NPs by ionotropic gelation for the controlled release of recombinant human erythropoietin (rHu-EPO). The size of the developed NPs ranged from 200 to 1000 nm. The encapsulation efficiency of the rHu-EPO was approximately 47.97% ± 4.10%. In addition, approximately 50% of the encapsulated rHu-EPO was released over two weeks in a sustained manner [151]. It has been reported that the prepared NPs were nontoxic to L929 cells. Moreover, ovalbumin was used as a model protein, and the loading efficiency of the ovalbumin varied from 4% to 17% [152]. Cross-linked carrageenan nanogels were prepared using a microemulsion method. The size of the NPs was smaller than 100 nm [153]. Chitosan–carrageen–TPP NPs by ionic gelation were developed [149,154]. The size of the NPs was approximately 150–300 nm [149,154]. Other carrageenan-based NPs for DDS have also been reported [155,156,157].

**Table 5:** Carrageenan NP production methods and delivery systems.

Serial number	Materials	Method	Particle size	Drug	References
1	Chitosan–carrageenan NPs	Ionotropic gelation	200 to 1000 nm	rHu-EPO	[151]
2	Chitosan/carrageenan	Ionic complexation	350–650 nm	Ovalbumin	[152]
3	Cross-linked–carrageenan NPs	Reverse microemulsion	100 nm	Methylene blue	[153]
4	Chitosan/carrageenan/TPP	Ionic gelation	150–300 nm	BSA	[149,154]
5	Carrageenan/protamine	Self-assembled	100–150 nm	NA	[155]
6	Carboxymethyl chitosan and carrageenan	NA	NA	Riboflavin	[156]
7	Carrageenan hydrogel	Gelation	NA	Methylene blue	[157]

## 6. Fucoidan NPs

**Figure 8:**
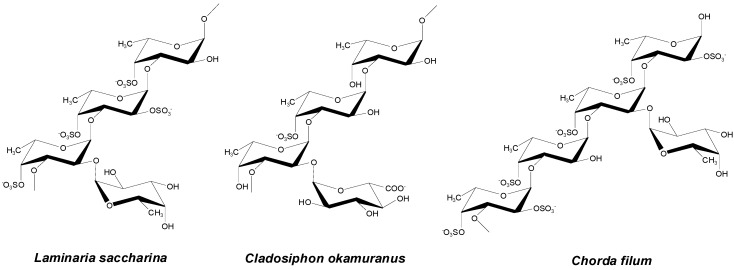
Structures of fucoidan. The figure was adopted with permission from [158].

Fucoidan is an anionic, sulfated polysaccharide found in brown seaweed (e.g., *Laminaria japonica*, *Macrocystis pyrifera*, *Fucus vesiculosus*, and *Ascophyllum nodosum*). It is mainly composed of α-(1-3)-linked fucose units or repeating disaccharide units of α-(1-3)- and α-(1-4)-linked fucose residues with *O*-2 branches (Figure 8). It has excellent bioactivity, including antivirus, antitumor, antithrombotic, anticoagulant, anti-inflammatory, and antioxidant activity [158,159,160,161]. Research on fucoidan for biomedical applications is still at the early stage of determining its exact function [162,163,164,165]. Some studies have been conducted regarding fucoidan-based NPs for the delivery of curcumin, doxorubicin, and growth factors.

### 6.1. Production of Fucoidan NPs

**Figure 9:**
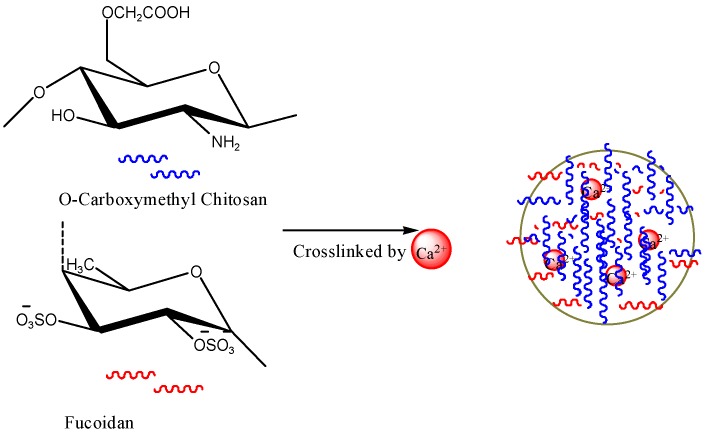
The formation of fucoidan NPs. The figures were adopted and redrawn from [172].

Chitosan/fucoidan-based NPs were synthesized using different types of methods, such as self-assembly, coacervation, polyelectrolyte complexing, ionic cross-linking, chemical modification, and emulsion (Table 6). Pinheiro *et al.* (2014) developed chitosan-fucoidan NPs using self-assembly for the delivery of bioactive compounds [166]. Lee and Lim *et al.* (2014) discussed the formation of chitosan-fucoidan NPs in two papers in detail [167,168]. The size of the developed chitosan–fucoidan NPs ranged from approximately 365–900 nm. A 1:1 ratio of chitosan to fucoidan was the optimum condition to produce NPs with a small size, high yield, and good stability. They also found that pH 5 was optimum to produce the polyelectrolyte NPs [167,168]. Kimura *et al.* [169] developed fucoidan-based NPs and assessed their activity against osteosarcoma. The experimental results suggested that the fucoidan NPs were more effective than native fucoidan [169]. Fucoidan nanogels with a particle size of approximately 123 nm were produced and used for cancer research [170]. Stable chitosan–fucoidan NPs encapsulating basic fibroblast growth factor (bFGF) were developed for nerve tissue engineering [171]. The particles were able to protect bFGF from degradation by enzymes. The particles were stable for a period of eight days. O-carboxymethyl chitosan/fucoidan NPs were prepared by ionic crosslinking and used for curcumin delivery [172] (Figure 9). The synthesized curcumin-loaded chitosan/fucoidan NPs dramatically increased the cellular uptake of curcumin. Fucolidan NPs by coacervation process and anionic emulsion polymerization were also developed [173,174].

**Table 6:** The production of fucoidan NPs.

Serial number	Materials	Method	Particle size	References
1	Chitosan-fucoidan NPs	Self-assembled	365–900 nm	[167,168]
2	Fucoidan lipid NPs	Chemical modification	100 nm	[169]
3	Fucoidan nanogels	Graft with hexadecylamine	123 nm	[170]
4	Chitosan-fucoidan	Coacervation process	154 and 453 nm	[173]
5	Fucoidan-coated poly(isobutylcyanoacrylate) NPs	Anionic emulsion polymerization	193 ± 4 nm to 399 ± 0.7 nm	[174]

### 6.2. Fucoidan NPs for Growth Factor Delivery

A diverse set of fucoidan NPs for the delivery of growth factors has been reported (Table 7). Huang *et al.* developed chitosan fucoidan-based NPs as vehicles for stromal cell-derived factor-1 (SDF-1) [175]. Chitosan–TPP–fucoidan NPs were developed using ionic gelation and PEC methods. The encapsulation efficiency of the chitosan-TPP-fucoidan NPs with SDF-1 was 60%–68%. The developed NPs showed a spherical diameter of approximately 173–403 nm. The amount of released SDF-1 from the chitosan-TPP-fucoidan NPs ranged from 17 to 23 ng/mL [175]. In work from the same group, chitosan-fucoidan NPs were produced by a PEC process and used for nerve tissue engineering. The size of the NPs was approximately 200 nm. The developed chitosan-fucoidan NPs were nontoxic to PC12 cells at a concentration of 125 ng/mL. Fucoidan-chitosan NPs were also prepared by a PEC processs with sonication [176]. BSA-loaded fucoidan-chitosan NPs showed a sustained release of BSA.

**Table 7:** Fucoidan NPs for growth factor delivery.

Serial number	Materials	Method	Size	Drug	References
1	Chitosan–fucoidan NPs	Polyelectrolyte complexing	200 nm	bFGF	[171]
2	Chitosan–TPP–fucoidan	Ionic gelation and polyelectrolyte complexing	173–403 nm	SDF-1	[175]
3	Fucoidan–chitosan NPs	Polyelectrolyte complexing	860 nm	BSA	[176]

### 6.3. Fucoidan NPs for Cancer Drug Delivery

**Figure 10:**
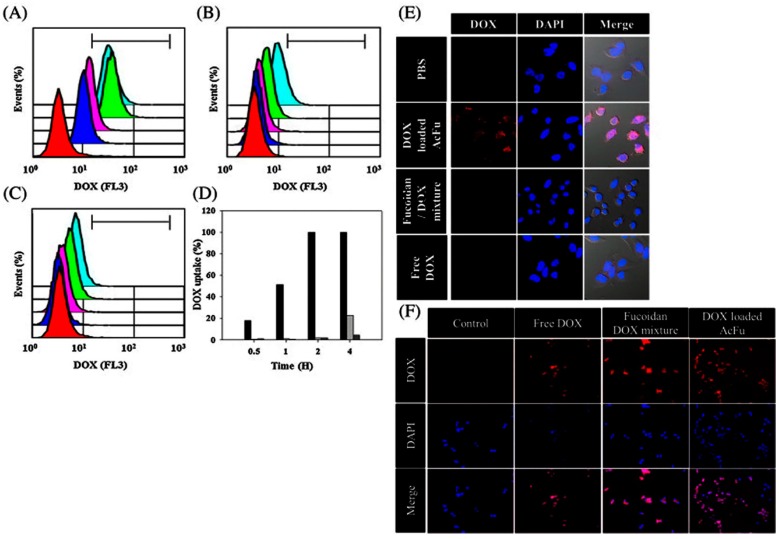
The time-dependent cellular uptake efficiency of doxorubicin was estimated by FACS analysis. Flow cytometry analysis of cells treated with (**A**) doxorubicin-loaded acetylated fucoidan NPs (AcFu NP); (**B**) natural fucoidan–doxorubicin mixtures; and (**C**) free doxorubicin. The colors in these graphs indicate the time after sample treatment: red—control; blue—30 min; pink—1 h; green—2 h; and sky blue—4 h. The uptake efficiencies at each time point are indicated by the bar graph in (**D**); (Black: doxorubicin-loaded AcFu NPs; gray: natural fucoidan–doxorubicin mixture; dark gray: free doxorubicin.); (**E**) Confocal images of doxorubicin uptake 4 h after treatment; (**F**) Confocal images of doxorubicin uptake in HCT-116 cells 4 h after sample treatment. The figures were adopted with permission from [191]. Copyright 2013, Elsevier.

A number of studies have reported that fucoidan itself has the capability of eliminating cancer cells by inducing apoptosis [177,178,179,180,181,182,183,184]. Therefore, various fucoidan-based NPs encapsulating anticancer drugs have been intensively developed in the pursuit of efficient cancer therapies (Table 8). Huang *et al.* (2011) developed chitosan-fucoidan NPs by ionic gelation for curcumin delivery [185]. Curcumin can be used as a natural anticancer drug, but its application has been hindered due to low bioavailability[186]. To improve bioavailability, curcumin-loaded NPs have been attempted [187,188,189]. The encapsulation efficiency of curcumin in chitosan-fucoidan NPs was higher than 85%. The release of curcumin increases with increasing pH; while the release of curcumin from the chitosan-fucoidan NPs was inhibited at pH 1.2, its release was increased at pH 6.0 and 7.0 [185]. In work from the same group, fucoidan NPs were developed using o-carboxymethyl chitosan for curcumin delivery. Ionic cross-linking has been used to produce these NPs. The encapsulation efficiency increased significantly to 92.8%. Curcumin was efficiently released from the chitosan-fucoidan NPs in a pH-dependent manner. While the release of curcumin was effective at pH 7.4, the release of curcumin was minimal at pH 2.5 [172,190]. Fucoidan NPs encapsulating DOX were also developed for cancer therapy [191]. The drug encapsulation efficiency was found to be 71.1% and 3.6%. The particle size was approximately 140 nm [191]. In HCT-8 cells (MDR model cells) exposed to DOX-loaded AcFu NPs, a time-dependent cellular internalization of the drugs was observed. Over 99% of the total DOX load was internalized by the HCT-8 cells after 2 h, whereas 1.99% and 1.79% of a fucoidan–DOX mixture and free DOX were internalized, respectively (Figure 10A–D). Only the DOX-loaded AcFu NPs could be clearly identified in confocal images (Figure 10E). In HCT-116 cells (non-MDR cells), the cellular uptake of free DOX was similar to that of the AcFu nanoparticle-encapsulated DOX (Figure 10F). However, these researchers mentioned that the mechanism behind this result was unclear mechanism (Figure 10 and Table 8).

**Table 8:** Fucoidan NPs for cancer drug delivery.

Serial number	Materials	Method	Particle size	Drug	References
1	Chitosan–fucoidan NPs	Self-assembled	Approximately 100 nm	PLL	[166]
2	*O*-carboxymethyl chitosan/fucoidan	Ionic cross-linking	270 nm	Curcumin	[172]
3	Chitosan–fucoidan	Ionic gelation	173 nm	Curcumin	[185]
4	Fucoidan NPs	Self-assembly	140 nm	Doxorubicin	[191]

## 7. Future Research in Seaweed Polysaccharide NPs

Ionic gelation and PEC methods provide excellent opportunities to produce large amounts of natural polymer-based NPs. However, there are several factors to be considered for developing natural polymer-based NPs, including the molecular weight of the polymers, addition time, pH, stirring speed, and temperature. To date, few *in vitro*, *in vivo* studies, and particle formation studies have been performed using alginate, carrageenan, and fucoidan NPs for drug delivery. There is a need for more *in vivo* research on carrageenan NPs and fucoidan NPs for further commercialization and use in clinical settings [192].

### 7.1. Active Targeting Molecules

Proper NP charge, size, and shape can improve drug delivery efficacy. In addition to those factors, engineering NPs with targeting moieties can significantly enhance drug delivery efficacy through the high accumulation of drugs in the targeted disease areas. In recent years, various targeting moieties, including peptides, small molecules, and polysaccharides themselves, have been incorporated into polysaccharide-based NPs to obtain targeted delivery. Somatostatin receptors, A54 hepatocarcinoma binding peptide, RGD peptide, and small molecules (e.g., glycyrrhetinic acid and vitamin E succinate) have also been used as targeting moieties [40]. Polysaccharides such as chitosan have also been known to have a capacity to promote drug absorption in the small intestine due to mucoadhesion [40,193,194,195,196,197].

### 7.2. Other Seaweed Polysaccharides

Future research can be focused on the formation of NPs from other seaweed polysaccharide-based biomaterials, such as ulvan and laminarin. Different seaweed polysaccharides have their own merits and applications. Ulvan is an anionic polysaccharide and thus easily forms NPs with cationic polymers such as chitosan, which indicates its potential as a biocompatible drug delivery carrier [198,199,200,201].

The seaweed polysaccharide NP preparations in this review were mainly based on combinations of chitosan and polyanions (e.g., alginate, carrageenan and fucoidan). The main reason to combine the chitosan and polyanions is to produce stable polymeric NPs, which can be achieved by the opposite charge interactions of chitosan and alginate. Developed NPs have been shown to protect the encapsulated materials and release drugs sustainably and effectively. Further advantages of the chitosan-polyanionic system include nontoxicity, biocompatibility and biodegradability [202].

## 8. Conclusions

In this review, we have discussed the production of various NPs using seaweed-based polysaccharides and their applications in drug delivery. The formation of seaweed polysaccharide-based NPs can easily be achieved by means of ionic gelation and PEC; these materials have the capacity to hold drug molecules and release them in specific locations. We believe that these methods will be increasingly utilized for the production of polysaccharide-based NPs in the future. Seaweed polysaccharide-based NPs have shown promising results in delivering proteins, peptides, anti-cancer drugs, and other drugs with increased bioavailability and sustained release properties. In particular, alginate-based NPs have extensively been studied for the delivery of anti-cancer drugs. In the last three decades, several studies have been conducted on seaweed polysaccharides both *in vitro* and *in vivo*; these studies have demonstrated the high stability and biocompatibility as well as sustained drug release achievable by these systems, which will support their future use in clinical settings. The introduction of targeting moieties to polysaccharide-based NPs will improve their therapeutic efficacy while also reducing undesired side effects.
